# Effects of Microtopography on Soil Microbial Community Structure and Abundance in Permafrost Peatlands

**DOI:** 10.3390/microorganisms12050867

**Published:** 2024-04-26

**Authors:** Man Zhang, Lingyu Fu, Dalong Ma, Xu Wang, Anwen Liu

**Affiliations:** 1 College of Geographical Sciences, Harbin Normal University, Harbin 150025, China; zhangman0018@163.com (M.Z.); m18846751794@163.com (L.F.); wx18800469360@163.com (X.W.); liuanwen0705@163.com (A.L.); 2 Heilongjiang Wuyiling Wetland Ecosystem National Observation and Research Station, Yichun 153000, China

**Keywords:** microtopography, microbial community, permafrost, peatland, diversity

## Abstract

Soil microorganisms play crucial roles in the stability of the global carbon pool, particularly in permafrost peatlands that are highly sensitive to climate change. Microtopography is a unique characteristic of peatland ecosystems, but how microtopography affects the microbial community structures and their functions in the soil is only partially known. We characterized the bacterial and fungal community compositions by amplicon sequencing and their abundances via quantitative PCR at different soil depths in three microtopographical positions (hummocks, flats, and hollows) in permafrost peatland of the Greater Xing’an Mountains in China. The results showed that the soil of hummocks displayed a higher microbial diversity compared to hollows. Microtopography exerted a strong influence on bacterial community structure, while both microtopography and soil depth greatly impacted the fungal community structure with variable effects on fungal functional guilds. Soil water content, dissolved organic carbon, total phosphorus, and total nitrogen levels of the soil mostly affected the bacterial and fungal communities. Microtopography generated variations in the soil water content, which was the main driver of the spatial distribution of microbial abundances. This information stressed that the hummock–flat–hollow microtopography of permafrost peatlands creates heterogeneity in soil physicochemical properties and hydrological conditions, thereby influencing soil microbial communities at a microhabitat scale. Our results imply that changes to the water table induced by climate warming inducing permafrost degradation will impact the composition of soil microbes in peatlands and their related biogeochemical functions, eventually providing feedback loops into the global climate system.

## 1. Introduction

Peatlands store approximately 528–600 Pg of organic carbon, accounting for 30% of the global soil carbon pool, although they only cover 3% of the global terrestrial area [1]. Peatlands are extensively water-saturated and anoxic environments, providing stable carbon reservoirs that act as one of the most important sinks of atmospheric CO_2_ [2]. Peatlands are mostly present in the middle and high latitudes of the Northern Hemisphere, where more than half are found in permafrost zones [3,4]. An interdependent relationship exists between permafrost and peatlands, whereby cryogenic permafrost conditions protect peat carbon from decomposition and prevent CO_2_ release into the atmosphere [5]. However, in the past 30 years, the increase in local temperatures of high-latitude permafrost regions was twice that of the global average, resulting in rapid permafrost thawing, which is a severe threat to peatland carbon stability [6,7]. Permafrost degradation not only significantly impacts the function of peatland ecosystems, leading to changes in hydrology, topography, vegetation composition, and biogeochemical cycling, but some of these changes also have the potential to induce substantial positive feedback within the climate system, which intensifies global warming even more [8].

A distinctive characteristic of permafrost peatland is its microtopography, characterized by hummocks, flats, and hollows, which are formed by frequent freezing and thawing of the soil’s active layer, combined with seasonal waterlogging and a particular vegetation cover. This microtopography creates habitats that are heterogeneous for hydrothermal conditions, vegetation cover, soil nutrient availability, and microclimate conditions [9,10]. Hummocks are raised structures that usually remain dry, as their elevated surfaces are relatively high with respect to the water table, while the surfaces of moist flats are relatively close to the water table, and depressed hollows are frequently inundated. The degradation of peatland permafrost due to global warming leads to alterations in this microtopography with changes in local hydrological processes and in successional plant communities, all of which affect the complexity and heterogeneity in these small-scale environments, with potentially profound impacts on peatlands [11,12]. Surveys of vegetation coverage sufficiently assess aboveground effects, but belowground effects are more difficult to capture. For this, the measurements of microbial communities can provide information about important belowground processes. Thus, the analysis of the patterns of spatial variability in soil microbial communities along small-scale environmental gradients is urgently needed to elucidate belowground mechanisms driving carbon sequestration of permafrost peatlands.

Peatlands contain a diverse array of microorganisms with variable capacity to use carbon sources and other substrates, thereby fulfilling various functional roles within peatland ecosystems [13]. Bacteria together with fungi account for over 90% of the total microbial biomass in soil and represent important players that control the biogeochemical cycling of soil nutrients [14]. While fungi are essential to decompose complex organic substrates, bacteria exhibit superior capability to utilize simple compounds [15]. Bacteria and fungi display disparate responses to changes in microhabitat, whereas shifts in the composition of their local microbial communities and their metabolic rates can dramatically impact the carbon dynamic in permafrost peatland [16]. Previous research has established that permafrost thaw causes a net loss of soil organic carbon in peatlands due to inadequate surface accumulation rates to compensate for deep organic carbon losses during thawing, which also highlights the importance of spatial heterogeneity in the carbon budget estimation of high-latitude permafrost peatlands [17]. The microtopography of peatland affects soil organic carbon components, soil fauna, enzyme activity, microbial biomass, and microbially regulated greenhouse gas emission processes [18,19,20]. Moreover, microtopography can mediate soil hydrothermal processes and nutrient movements that may further affect microbial community composition and diversity in the soil [21]. Although studies have been conducted in palustrine forested wetlands, boreal peatlands, and the Qinghai-Tibetan Plateau, there is much less information available about the relationships between microbial community compositions affected by microtopography in permafrost peatlands [22,23,24]. Considering the huge carbon storage in permafrost peatlands, a better understanding of the diversity and distribution of microbial communities in each microtopographical position will assist in more accurately simulating and predicting the role of peatlands on global carbon balance.

The permafrost peatland of the Greater Xing’an Mountains in China, situated at the southern edge of the high-latitude permafrost zones of Eurasia, is particularly sensitive to climate change and is at risk of transitioning from a carbon sink to a carbon source [25]. Thawing of permafrost peatland not only creates a deeper and warmer active layer but also results in the collapse of the local land surface and induces substantial changes in the peatland structure and biogeochemical functions [26]. A significant influence of geomorphic disturbances on the storage capacity of soil organic carbon has been demonstrated, with major differences in soil organic carbon content between hummocks and hollows [27]. The significance of microtopography in controlling carbon and nutrient circulation in permafrost peatlands is emphasized here. With increasing peatland depth, a decrease in oxygen combined with an increasing distance to fresh plant-derived debris provides favorable conditions for anaerobic microbial communities [28]. However, in recent years, global warming has become the driving force behind the declining water tables in peatlands, which has led to a shift from an anaerobic soil environment of peatland to an aerobic one [29]. Therefore, it is anticipated that the microbial community composition and activity will be significantly influenced by microenvironmental changes. To fill in the existing knowledge gap about the relationship between soil microbial communities in permafrost peatlands and microtopography and depth, we examined various depths (0–20 cm, 20–40 cm, and 40–60 cm) in three microtopographical positions (hummocks, flats, and hollows) and determined their variations in bacterial and fungal community structures and abundances in the Greater Xing’an Mountains permafrost peatland. We hypothesized that (1) there is spatial variability in the microbial community composition in the three microtopographical positions; (2) microbial diversity and abundance within each microtopographical position decreases with depth; (3) spatial heterogeneity of soil properties caused by microtopography and depth can explain most variations in the microbial community.

## 2. Materials and Methods

### 2.1. Description of the Selected Study Area

The study area is located at the Mohe Forest Farm in a high-latitude area with permafrost in the Greater Xing’an Mountains of China. This area has a cold temperate continental monsoon climate, with brief and moderate summers and extended, cold winters that have reached temperatures as low as −52.3 °C. Typically, there are 85 to 110 days without frost each year, with an annual rainfall of 450 to 550 mm. The investigated region has a distribution of peat, meadow, and swamp soil [30]. A large part of the area is covered with continuous permafrost, with a 70–160 cm thick active layer and a 10–90 cm thick peat layer. The peatland ground cover vegetation is dominated by *Sphagnum* mosses, interspaced with sedges (*Eriophorum vaginatum* and *Carex globularis*) and low-growing vascular plants (*Rhododendron tomentosum* and *Vaccinium uliginosum*).

### 2.2. Experimental Design and Soil Collection

Three 10 × 10 m typical ombrotrophic peatland locations with similar topography, hydrology, and vegetation conditions were selected as sampling plots. Sampling was performed in August 2020. In each plot, three microtopographies were selected randomly to represent three replicates, and each microtopography was divided into three positions (hummock, flat, and hollow) based on the depth of the water table and the presence of dominant bryophyte species. In each sampling plot, a PVC pipe was inserted to monitor changes in water table depth. Hummocks were characterized by an average water table at a distance of −35.9 ± 2.6 cm from their surface during the sampling period and were dominated by *Sphagnum magellanicum* and *Sphagnum palustre*; flats had an intermediate average water table (−16.2 ± 0.7 cm) and were dominated by *Sphagnum fuscum* and *Sphagnum nemoreum*; and hollows with a shallow average water table (−9.8 ± 1.1 cm) were dominated by *Sphagnum recurvum*. The hummocks had a mean height of 24.7 cm and a mean basal area of 595.6 cm^2^. In each selected position, soil was collected at a depth of 0–20 cm, 20–40 cm, and 40–60 cm using a 5 cm-wide peat auger. Triplicate samples taken at the same depth within a microtopography were thoroughly mixed to form a composite sample, resulting in 27 composite samples (3 microtopographies × 3 depths × 3 plots). These soil samples were transported to the lab in sterile zipper bags at 4 °C. There, stones and rough plant material were removed, and the soil was passed through a 2 mm sieve. One part of the sieved soil was air-dried and used for the determination of physicochemical properties. The remaining portion was stored at −80 °C prior to DNA extraction.

### 2.3. Determination of Soil Properties

The pH of the soil was measured using a pH meter (PHS-25, Shanghai, China) at a 1:2.5 ratio of soil to water mass. Molybdenum antimony blue colorimetry was used to determine the total phosphorus (TP) amount in the soil [31]. Soil total nitrogen (TN) was analyzed using an automatic Kjeldahl azotometer (K9860, Hanon, Jinan, Shandong, China) based on the Kjeldahl method [32]. Soil water content (SWC) was determined by weight loss after drying the soil at 105 °C for 24 h. A multi-N/C 3100 analyzer (Jena, Germany) was used to determine total organic carbon (TOC) and dissolved organic carbon (DOC) [33]. Nitrate nitrogen (NO_3_^−^-N) and ammonium nitrogen (NH_4_^+^-N) in the soil were measured after extraction with 2 M KCl using a continuous flow analyzer (Skalar SAN++, Breda, The Netherlands) [34].

### 2.4. Microbial DNA Extraction, Amplification, and Sequencing

Microbial DNA was extracted from every soil sample using the FastDNA spin kit (MP Biomedicals, Santa Ana, CA, USA). The purity of the resulting DNA was evaluated using a SmartSpec Plus spectrophotometer (Bio-Rad Laboratories, Hercules, CA, USA). PCR was used to amplify the bacterial 16S rRNA gene with the primer pair 338F/806R and the ITS region of the fungal gene with the primer pair ITS1F/ITS2R [35,36]. Thermocyling began with a 5 min denaturation at 95 °C, followed by 27 cycles for bacteria and 35 cycles for fungi. Each cycle included 30 s of denaturation at 95 °C, 30 s of annealing at 55 °C, and 45 s of extension at 72 °C. The process concluded with a final extension step lasting 10 min at 72 °C. Triplicate PCR products were pooled and purified with the QIAquick PCR Purification Kit (Qiagen, Hilden, Germany) and sequenced by Majorbio Biopharm Technology Co., Ltd. (Shanghai, China) using the Illumina MiSeq PE300 platform. The raw data were stored in the NCBI database (PRJNA1095832 and PRJNA1095852).

### 2.5. Quantitative PCR (qPCR)

The abundance of bacteria and fungi was assessed via qPCR using the primer pairs Eub338/Eub518 and ITS1F/ITS2R, respectively [37]. All amplifications were conducted in triplicates using an ABI 7500 system (Applied Biosystems, Foster, CA, USA). The qPCR conditions were as follows: 95 °C for 5 min, followed by 39 cycles of denaturation at 95 °C for 15 s, annealing at 58 °C (bacteria) or 55 °C (fungi) for 15 s, and extension at 72 °C for 30 s. All amplifications were performed in triplicates using an ABI 7500 system (Applied Biosystems, USA). A standard curve was constructed using serial 10-fold dilutions of a linearized plasmid. The determined amplification efficiencies were greater than 95% and the correlation coefficients were greater than 0.99.

### 2.6. Statistical Analysis

The primer fragments of the sequences were excised, and unmatched primer sequences were discarded using QIIME2 v.2020.2. Subsequently, quality control, denoising, splicing, and chimera removal were performed by DADA2. The resulting sequences were used to determine amplicon sequence variants (ASV) with DADA2 [38]. Taxonomic assignment was conducted by the SILVA v.138.1 database for bacteria and the UNITE v.8.0 database for fungi. Normalization was performed according to the lowest number of reads to achieve equal sequencing depth among samples. Rarefaction curves confirmed the achievement of adequate sampling depth across all samples, indicating that sequence reads were sufficient to capture a majority of diversity within the bacterial and fungal communities (Figure S1A,B). The number of ASVs was used to determine alpha diversity indices (ACE, Chao, and Shannon indices) by Mothur v.1.30.2. One-way analysis of variance (ANOVA) was conducted with SPSS 26.0 software to determine the significance (*p* < 0.05) of variations in soil physicochemical characteristics in different samples. The vegan package in R4.1.1 was used for non-metric multidimensional analysis (NMDS) and Adonis jointly to analyze the differences in the microbial community structure in three microtopographical positions [39]. The functional traits of the bacterial and fungal communities were predicted using PICRUSt2 and FUNGuild, respectively [40,41]. CANOCO 5.0 was utilized to analyze the correlation between soil physicochemical characteristics and microbial communities through redundancy analysis (RDA). Pearson correlations generated in R4.1.1 were used to visualize correlations between bacterial and fungal abundances and soil characteristics in a heatmap.

## 3. Results

### 3.1. Physicochemical Properties of the Soil

The pH of the collected soil from all three microtopographical positions was acidic. The pH varied with depth, first becoming more acidic and then increasing with depth (Table 1). The highest total phosphorus (TP) and total nitrogen (TN) were found in the soil at 20–40 cm, with significantly higher levels in hollows compared to hummocks, at all depths (*p* < 0.05). Hummocks had a significantly lower soil water content (SWC) than the other two positions (*p* < 0.05). The soil total organic carbon (TOC) content was significantly higher at a depth of 20–40 cm compared to 0–20 cm and 40–60 cm, and this applied to all three positions (*p* < 0.05). Soil-dissolved organic carbon (DOC) was significantly higher in hummocks compared to flats and hollows (*p* < 0.05). The content of nitrogen in the form of nitrate (NO_3_^−^-N) and ammonium (NH_4_^+^-N) decreased as depth increased across all three microtopographical positions.

### 3.2. Diversity of the Soil Bacterial and Fungal Communities

**Figure 1:**
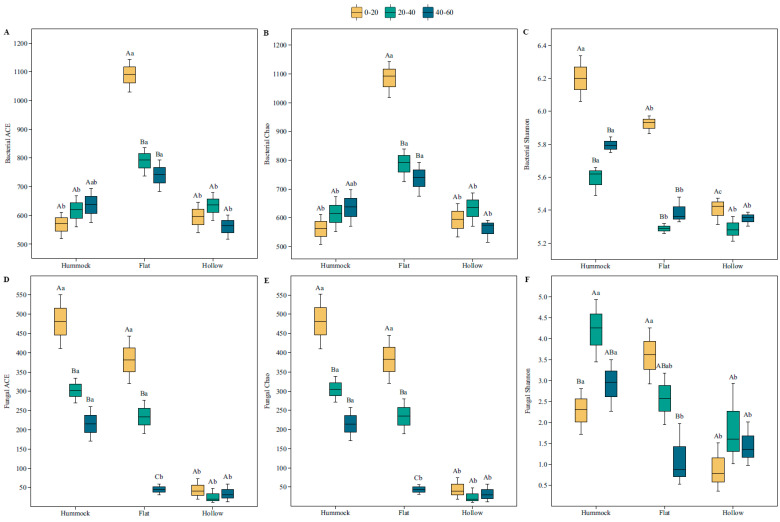
Boxplots of microbial alpha diversity: (**A**) bacterial ACE; (**B**) bacterial Chao; (**C**) bacterial Shannon; (**D**) fungal ACE; (**E**) fungal Chao; (**F**) fungal Shannon. Different capital letters indicate significant differences among three soil depths in a microtopographical position (*p* < 0.05), and different lowercase letters indicate significant differences among three microtopographical positions at the same soil depth (*p* < 0.05).

A total of 1,027,584 high-quality sequences targeting the 16S rRNA gene with a mean length of 416 bp were obtained for bacteria. A total of 1,220,376 high-quality sequences with a mean length of 261 bp were obtained for fungi (Table S1). Bacterial ACE and Chao indices reached maximum values in flats at 0–20 cm and were significantly higher in flats than in hollows in the three soil depths (*p* < 0.05). No significant differences were observed in the bacterial ACE and Chao indices for different depths of hummocks and hollows (*p* > 0.05, Figure 1A,B). The bacterial Shannon index was significantly lower in flats and hollows compared to hummocks (*p* < 0.05, Figure 1C). At 0–20 cm and 20–40 cm, fungal ACE and Chao indices were significantly higher in hummock and flat than in hollow (*p* < 0.05), but significantly higher indices were observed in hummock at a depth of 40–60 cm than in other positions (*p* < 0.05). Fungal ACE and Chao indices produced no significant differences among the assessed depths in hollows (*p* > 0.05), whereas they exhibited significant variations along the soil depths in flats (*p* < 0.05, Figure 1D,E). The fungal Shannon index was markedly higher in hummocks compared to hollows (*p* < 0.05), but it did not differ significantly with depths in hollows (*p* > 0.05, Figure 1F).

**Figure 2:**
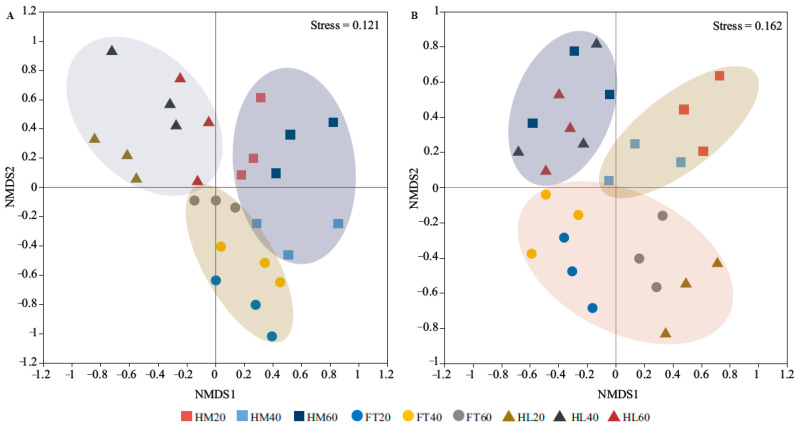
Non-metric multidimensional scaling analysis (NMDS) of soil microbial community: (**A**) bacterial community; (**B**) fungal community.

Non-metric multidimensional scaling analysis (NMDS) in accordance with the Bray–Curtis distance separated the bacterial communities of the different microtopographical positions (Figure 2A, stress = 0.121). The Adonis analysis further confirmed that microtopography (R^2^ = 0.581, *p* = 0.001) but not depth (R^2^ = 0.252, *p* = 0.079) affected the observed variation among bacterial communities. For fungal communities, soil samples at different depths in hummocks (HM) and flats (FT) formed distinct clusters, whereas the soil at a depth of 40–60 cm from flats (FT60) and 0–20 cm from hollows (HL20) clustered closely together. The Adonis analysis also showed that the detected fungal communities were driven by both microtopography (R^2^ = 0.433, *p* = 0.007) and depth (R^2^ = 0.319, *p* = 0.032, Figure 2B, stress = 0.162).

### 3.3. Structures and Compositions of the Soil Bacterial and Fungal Communities

**Figure 3:**
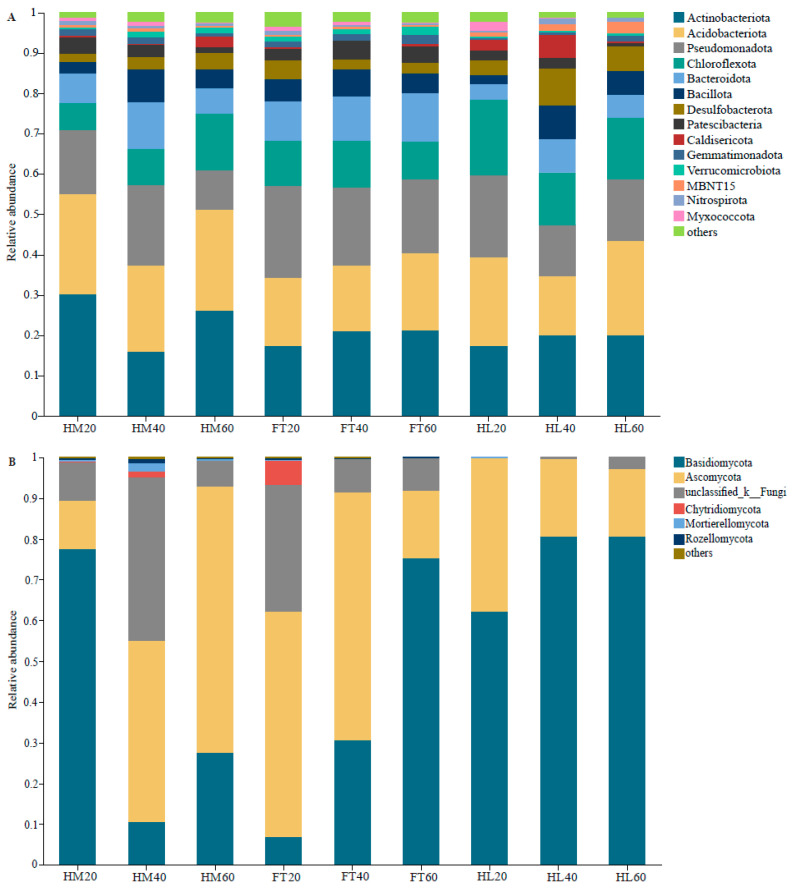
Characteristics of microbial community compositions at the phylum level: (**A**) bacterial phyla; (**B**). fungal phyla.

The bacterial dominant phyla in this permafrost peatland soil were identified as Actinobacteriota (16.11–30.19%), Acidobacteriota (14.68–25.04%), Pseudomonadota (9.75–22.87%), Chloroflexota (6.68–18.63%), and Bacteroidota (3.83–12.16%). There was a significantly higher relative abundance of Actinobacteriota in HM20 than in FT20 and HL20 (*p* < 0.05). The relative abundance of Acidobacteriota first decreased and then increased with depth in all three microtopographical positions. The relative abundance of Pseudomonadota showed an increasing and then decreasing trend in HM with depth, whereas an opposite trend was observed in HL, and Pseudomonadota decreased continuously with depth in FT. Chloroflexota displayed a significantly higher relative abundance in HL20 compared to HM20 and FT20 (*p* < 0.05). The relative abundance of Bacteroidota was markedly lower in HL20 and HL60 than in FT20 and FT60 (*p* < 0.05, Figure 3A). Of the identified fungal phyla, Basidiomycota (6.84–80.73%) and Ascomycota (11.89–65.30%) were dominant. The relative abundance of Basidiomycota was markedly higher in HM20 compared to FT20, whereas it was significantly lower in HM40 and HM60 compared to HL40 and HL60 (*p* < 0.05). At depths of 0–20 cm and 20–40 cm, the relative abundance of Ascomycota reached the highest value in FT, but the highest value at 40–60 cm depth was detected in HM (Figure 3B).

**Figure 4:**
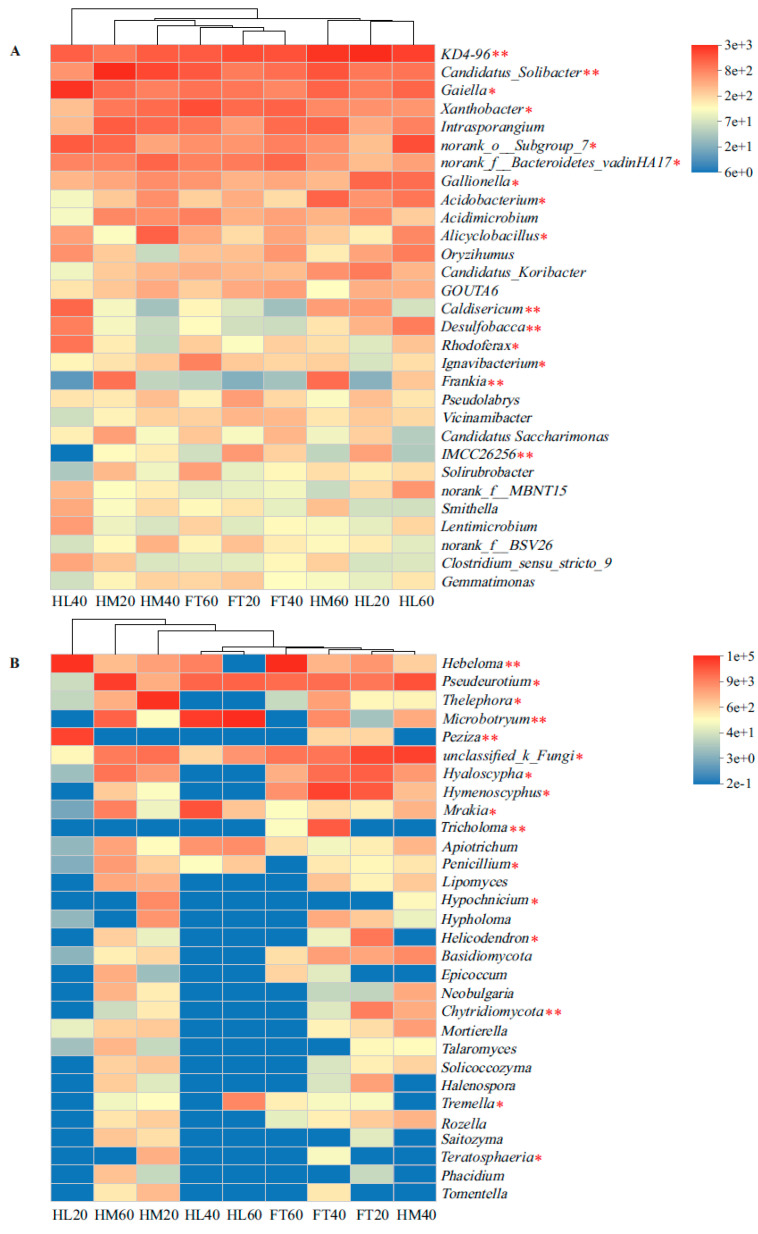
Heatmaps of the top 30 microbial composition differences at the genus level: (**A**) bacterial genera; (**B**) fungal genera. Significance levels were labeled as follows: *, *p* < 0.05; **, *p* < 0.01.

At the genus level of bacteria, the relative abundance of *IMCC26256* (0.03–2.66%), *Frankia* (0.05–5.19%), *Ignavibacterium* (0.31–3.82%), *Rhodoferax* (0.23–4.68%), *Desulfobacca* (0.24–4.12%), *Caldisericum* (0.13–5.66%), *Alicyclobacillus* (0.54–6.12%), *Acidobacterium* (0.48–5.96%), *Gallionella* (1.65–5.65%), *norank_f__Bacteroidetes_vadinHA17* (1.57–5.76%), *norank_o__Subgroup_7* (1.49–8.05%), *Xanthobacter* (1.47–8.25%), *Gaiella* (3.51–5.97%), *Candidatus_Solibacter* (2.87–8.94%), and *KD4-96* (4.37–12.99%) produced significant differences in different microtopographical positions and soil depths (*p* < 0.05, Figure 4A). At the fungal genus level, the relative abundances of *Teratosphaeria* (0–1.22%), *Tremella* (0–4.46%), *Chytridiomycota* (0–5.86%), *Helicodendron* (0–7.58%), *Hypochnicium* (0–3.92%), *Penicillium* (0–2.27%), *Tricholoma* (0–17.84%), *Mrakia* (0–23.70%), *Hymenoscyphus* (0–37.68%), *Hyaloscypha* (0–15.94%), *unclassified_k_Fungi* (0.11–31.07%), *Peziza* (0–37.27%), *Microbotryum* (0–71.56%), *Thelephora* (0–65.28%), *Pseudeurotium* (0.02–44.09%), and *Hebeloma* (0–62.23%) varied markedly at different depths in the three microtopographical positions (*p* < 0.05, Figure 4B).

### 3.4. Bacterial and Fungal Abundances

**Figure 5:**
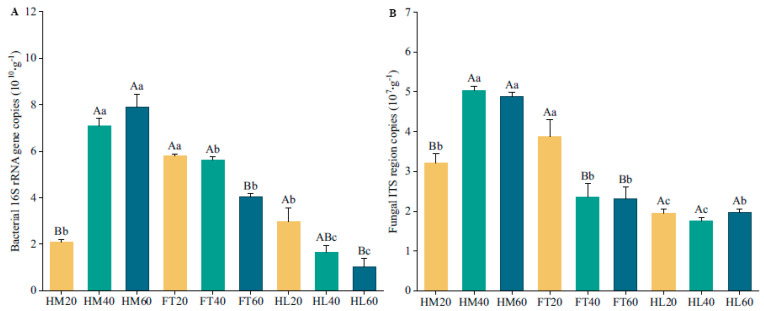
Bar plots of microbial abundances based on qPCR: (**A**) bacterial abundance; (**B**) fungal abundance. Different capital letters indicate significant differences among three soil depths in a microtopographical position (*p* < 0.05), and different lowercase letters indicate significant differences among three microtopographical positions at the same soil depth (*p* < 0.05).

This revealed a continuous increase in bacterial abundance with depth in HM, but the opposite trend was observed in FT and HL. At 20–40 cm and 40–60 cm depth, bacterial abundance was significantly higher in HM than in FT and HL (*p* < 0.05), whereas at 0–20 cm, it was significantly higher in FT than in HM and HL (*p* < 0.05, Figure 5A). Fungi showed a significantly higher abundance in FT compared to HM and HL at 0–20 cm, but exhibited a markedly lower abundance in FT and HL compared to HM at 20–40 cm and 40–60 cm (*p* < 0.05). There was a significantly lower abundance of fungi at 0–20 cm compared to other depths in HM (*p* < 0.05), whereas FT20 produced the highest fungal abundance in FT. There were no significant differences in fungal abundance between the depths of HL (*p* > 0.05, Figure 5B).

### 3.5. Functional Metabolic Pathways

**Figure 6:**
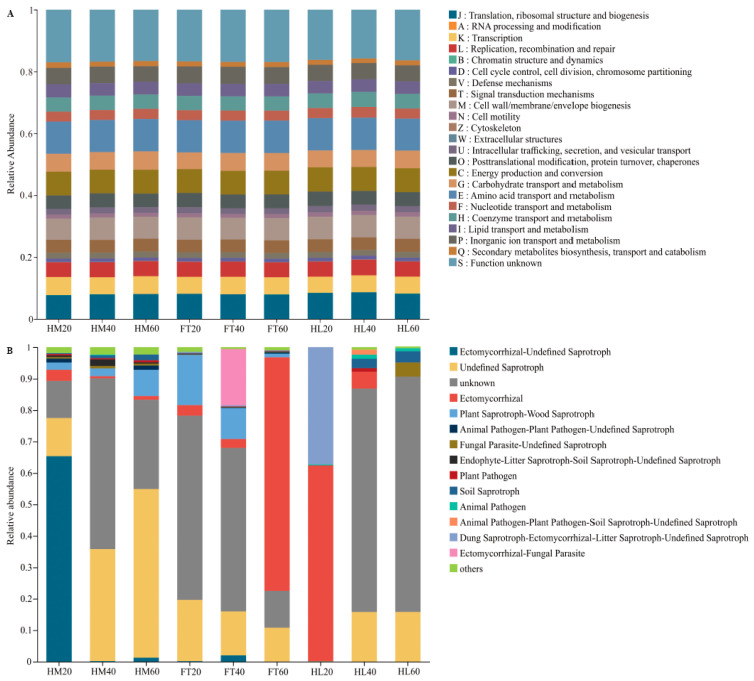
Bacterial functional groups inferred by PICRUSt2 and fungal functional guilds inferred by FUNGuild: (**A**) bacterial functional groups; (**B**) fungal functional guilds.

The PICRUSt2 software v2.3.0 was used to predict the functional gene compositions of bacterial communities based on Cluster of Orthologous Groups (COG) databases. The main functions of the bacterial community were amino acid transport and metabolism, translation, ribosomal structure and biogenesis, and energy production and conversion. The categories and abundance of bacterial community functions varied little with depth in the three microtopographical positions (Figure 6A). The fungal functional guilds were performed by FUNGuild analysis, dominated by undefined saprotroph, ectomycorrhizal, and ectomycorrhizal-undefined saprotroph. The results showed that the relative abundance of undefined saprotroph was higher in HM than in FT and HL at 20–40 cm and 40–60 cm depth. At 0–20 cm, the highest abundance of undefined saprotroph was found in FT. Ectomycorrhizal showed a higher abundance in FT60 and HL20 and ectomycorrhizal-undefined saprotroph reached the highest abundance in HM20 (Figure 6B).

### 3.6. Effects of Environmental Factors on the Community Structures and Abundances

**Figure 7:**
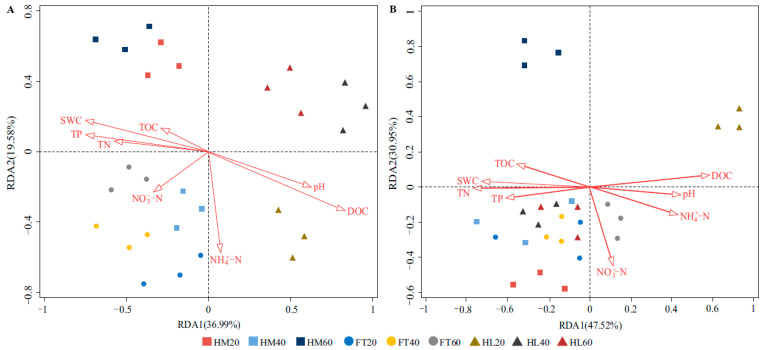
Redundancy analysis (RDA) of microbial community structures and environmental factors in different positions: (**A**) bacterial community; (**B**) fungal community.

RDA analysis of the bacterial findings indicated that the two axes explained 36.99% and 19.58% of the changes in the bacterial community structure, respectively. Among these factors, the distribution of the bacterial community was significantly affected by DOC (F = 2.9, *p* = 0.010), SWC (F = 2.2, *p* = 0.026), and TP (F = 2.1, *p* = 0.048, Figure 7A). The RDA of fungal results revealed that the cumulative variance contribution of the two axes was 78.47%. Here, DOC (F = 2.6, *p* = 0.018), TN (F = 1.9, *p* = 0.026), and SWC (F = 1.8, *p* = 0.028) were the main environmental factors influencing the structure of the fungal community (Figure 7B).

**Figure 8:**
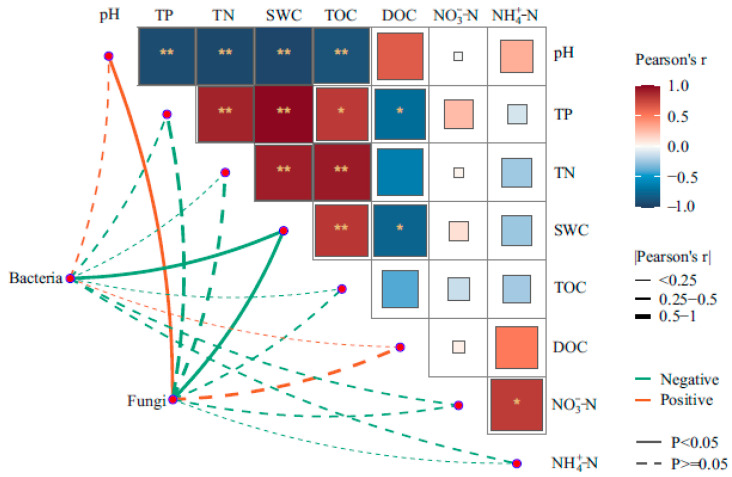
Pearson’s test for correlations among bacterial and fungal abundances and physicochemical properties of the soil. Significance levels were labeled as follows: *, *p* < 0.05; **, *p* < 0.01.

Pearson’s correlation analysis indicated that the bacterial abundance negatively correlated with SWC (r = −0.700, *p* = 0.036), whereas a positive correlation was found for the fungal abundance with pH (r = 0.695, *p* = 0.039) and a negative correlation with SWC (r = −0.743, *p* = 0.022, Figure 8).

## 4. Discussion

### 4.1. Effects of Microtopography and Depth on Microbial Diversity

The results indicated that microtopography exerted a significant influence on the α and β diversity of bacteria and fungi, and their diversity also differed with soil depth. Jaatinen et al. [42] demonstrated that the diversity of microbial communities in boreal peatlands strongly depended on peatland types and were not only related to water table depths but also to the plant community composition. We found a markedly higher diversity of bacteria and fungi in hummocks compared to hollows. The hummock–hollow microtopography can create a fertile island effect, with hummocks being drier and warmer with lower water tables than hollows, enabling more plant growth, and favoring microbial activity, including aerobic decomposition of soil organic matter [43]. The higher soil water content of hollows creates anoxic conditions that may suppress microbial decomposition of organic matter [44]. The bacterial richness was significantly higher in flats compared to hummocks and hollows, while fungi produced significantly higher richness in hummocks and flats than in hollows at 0–20 cm and 20–40 cm depth. Accordingly, it is anticipated that the areas of high bacterial and fungal diversity in these permafrost peatlands might be most impacted by future potential water table drawdowns as a result of climate change scenarios. These communities may shrink or move downwards within the soil profile, and even slight modifications can have profound effects on greenhouse gas emission processes regulated by microorganisms.

The NMDS and Adonis analysis indicated that microtopography was the main factor responsible for bacterial community variability, while both microtopography and soil depth accounted for variation in fungal communities, supporting our first hypothesis. In general, bacteria exhibit a wide range of tolerance to moisture niches, and their diverse metabolic pathways allow a wider redox tolerance, whereas most fungi display intolerance to anoxic conditions and are strongly sensitive to changes in soil moisture; thus, the vertical pattern of the fungal community might have correlated with the redox potential and soil organic matter quality [45]. The hummocks of the investigated site were elevated 24.7 cm above the soil surface and provided adequate oxygen supply, facilitating the growth of *Carex globularis* and *Sphagnum magellanicum* as well as sparse dwarf shrubs such as *Vaccinium uliginosum*. Hollows and flats were 6.6–22.5 cm above the water table, exhibiting higher water contents, and were mainly colonized by *Sphagnum recurvum* and *Eriophorum vaginatum* with *Sphagnum fuscum*. Chroňáková et al. [13] also revealed that peatland microhabitats dominated by distinct vascular plants harbored discrete microbial communities. Moreover, changes in the water table may affect plant communities with cascading effects on microbial community composition. Therefore, the heterogeneous habitat formed by the spatial variation in the hummock–flat–hollow microtopography produces different litter types and root exudates from distinct *Sphagnum* mosses and sedges, and this supplied a range of resources with varying rates of decomposition, potentially impacting the makeup of the soil microbial community and its related biogeochemical functions.

### 4.2. Changes in Microbial Community Structures in Different Positions

Our study revealed that Actinobacteriota was the dominant bacterial phylum (16.11–30.19%) in all three microtopographical positions. Actinobacteriota members are crucial decomposers of lignocellulosic plant litter. The relative abundance of Actinobacteriota at a depth of 0–20 cm was significantly higher in hummock compared to flat and hollow soil, potentially due to the presence of woody shrubs (*Vaccinium uliginosum* and *Rhododendron tomentosum*) [46]. Acidobacteriota members are generally oligotrophic and many prefer nutrient-poor environments [47]. Our observations showed that the relative abundance of Acidobacteriota was lower at a depth of 20–40 cm, where organic carbon content was higher compared to 0–20 cm and 40–60 cm, which is consistent with the results of Xiao et al. [48]. In comparison, the relative abundance of Chloroflexota is commonly present in oxygen-deficient environments with high soil moisture, and these bacteria play a vital role in breaking down complex polymeric organic compounds [49]. Members of Pseudomonadota are very diverse organisms that can adapt to a wide variety of lifestyles and wetland environments [50]. We found that the relative abundance of Pseudomonadota was higher in HM40, FT20, and HL20, which parallels previous studies that Pseudomonadota is primarily responsible for plant biomass degradation and nutrient cycling [51]. The phyla Basidiomycota and Ascomycota emerged as the predominant phyla of the fungal community, with the former being more specialized in lignin breakdown and the latter in cellulose and hemicellulose degradation [52]. It has been shown that the structure of a fungal community in boreal peatland is potentially shaped at the micro-scale by the water table level [53]. Our study is in agreement with Peltoniemi et al. [54] that fluctuations in the water table, changing oxygen availability, and redox potential favor Ascomycota while exerting detrimental effects on Basidiomycota. This may be due to Ascomycota having a greater ability to tolerate stress and use resources efficiently in harsh environments. Furthermore, stronger variations in the relative abundance of various fungal functional guilds were observed among the different soil layers in relation to microtopography as opposed to bacterial variation. Consequently, under the influence of climate change and permafrost degradation, alterations in microbial communities at the metabolic functional group level are anticipated to occur due to water table drawdowns, which would particularly affect fungi, and when the underlying peat layers become oxygenated, this could potentially facilitate the decomposition of frozen and new carbon, thereby contributing to positive feedback to climate change.

### 4.3. Variations in Microbial Abundances Response to Microtopography

The microtopographic heterogeneity of permafrost peatlands can create distinct microhabitats that represent aerobic and anaerobic environments. Soil water conditions act as an environmental filter, strongly influencing microbial survival, growth, and inter-species dynamics by regulating niche suitability and nutrient availability [55]. We found that bacterial and fungal abundances at different soil depths peaked in different microtopographical positions, with peaks in flats at a depth of 0–20 cm, but in hummocks at a depth of 20–40 cm and 40–60 cm, while the water table was approximately the same. These observations do not completely support our second hypothesis that microbial abundance within each microtopographical position decreases with depth. This suggests that the potential hotspot for abundance is likely near the boundary between aerobic and anaerobic layers, where the water table fluctuates [56]. Asemaninejad et al. [53] also observed the highest microbial abundances at intermedium moisture levels near the water table in peatland, which might have contained overlapping aerobic and anaerobic microbial communities. Hollows exhibited significantly higher levels of water saturation, and the limited availability of oxygen strongly suppressed microbial activity and proliferation [57]. Pearson correlation analysis showed that SWC was mostly shaping the vertical microbial abundance, which is consistent with other studies [58]. These findings highlight how important localized hydrological processes are to control the abundance of soil microbial communities.

### 4.4. Impacts of Environmental Factors on Microbial Community Structures

The reported RDA results indicated that DOC as well as SWC were the crucial factors that shaped the soil microbial community. DOC is more active in the carbon pool of peatlands and is readily assimilated and utilized by microorganisms [59]. Moreover, DOC not only constitutes a carbon source for microbial growth but also enhances the availability of other elements for microorganisms [60]. We found that hollows and flats have significantly lower DOC concentrations relative to hummocks, suggesting a difference in the biogeochemical milieu. The distribution of vegetation in each microtopographical position is closely related to the water table because vascular plants are better adapted to relatively dry and aerobic hummocks, and the litter of vascular plants can release significant amounts of DOC during rapid microbial decomposition [61]. SWC is considered the primary factor limiting the growth of microorganisms and as such it shapes the soil microbial community by controlling the aeration and oxygen content [62,63]. Liu et al. [64] reported that the spatial distribution of phosphorus in the soil strongly correlated with microtopography, and this also caused alterations in the structure of the bacterial community, which aligns with our findings. Furthermore, changes in soil fungal community structures were also related to TN. On the one hand, certain soil fungi can increase the content of nitrogen in the soil through the decomposition of plant litter [65]. On the other hand, mycorrhizal fungi transport nitrogen from the soil to surface plants in exchange for carbon that has been assimilated by plants [66]. Through incorporating these findings, our study showed that microtopography shapes the microbial community structures at a small scale, which is attributed to environmental heterogeneity in hydrological conditions and soil physicochemical properties, thereby confirming our third hypothesis.

## 5. Conclusions

In this study, the soil microbial community structures and abundances in three microtopographical positions at different soil depths were investigated. This approach contributes to a more comprehensive understanding of the critical process of adjustment and maintenance in the microhabitats of peatland ecosystems in the permafrost region of the Greater Xing’an Mountains. Our findings showed that elevated and drier hummocks exhibited a higher microbial diversity in comparison to depressed and wetter hollows. Microtopography plays a central role in driving soil bacterial and fungal community structures, while the fungal community was more sensitive to soil depths than that of bacteria. The relative abundance of fungal functional guilds also varied markedly along the soil profile. The vertical differences in microbial abundances within microtopography were driven by the soil water content. These results suggest that microtopography regulates the microbial community structure and microbially mediated biogeochemical processes by altering the soil properties and hydrologic conditions. This is important for peatlands at high latitudes, which act as sentinels of climate change, where small variations can alter the microorganism–carbon–climate feedback. Thus, there is a need to incorporate microtopographical characteristics into future climate modeling projections, which will be beneficial to improve the accuracy of simulating the effects of climate change on the carbon dynamics of permafrost peatlands.

## Figures and Tables

**Table 1:** Soil physicochemical properties at different soil depths in the three microtopographical positions.

Position	Soil Depth	pH	TP (g·kg^−1^)	TN (g·kg^−1^)	SWC (%)	TOC (g·kg^−1^)	DOC (g·kg^−1^)	NO_3_^–^-N (mg·kg^−1^)	NH_4_^+^-N (mg·kg^−1^)
Hummock	0–20	4.95 ± 0.01 Aa	2.14 ± 0.07 Bc	14.51 ± 0.72 Cb	58.15 ± 3.28 Bb	215.32 ± 22.15 Ba	0.41 ± 0.06 Aa	7.16 ± 1.09 Ab	72.57 ± 7.92 Aa
	20–40	4.76 ± 0.01 Ca	2.38 ± 0.13 Ac	20.27 ± 1.4 Ab	66.67 ± 4.91 Bb	319.63 ± 36.19 Ab	0.33 ± 0.05 Aa	5.32 ± 0.82 Ba	46.81 ± 5.21 Ba
	40–60	4.89 ± 0.01 Ba	2.34 ± 0.12 ABb	17.12 ± 1.19 Bb	69.22 ± 5.41 Ab	247.51 ± 26.31 Bb	0.12 ± 0.01 Ba	4.17 ± 0.72 Ba	32.15 ± 3.41 Cb
Flat	0–20	4.88 ± 0.01 Ab	2.53 ± 0.19 Bb	18.68 ± 1.24 Ba	71.14 ± 4.32 Ba	221.36 ± 24.24 Ca	0.15 ± 0.05 Ab	8.13 ± 1.21 Bab	65.88 ± 6.84 Aa
	20–40	4.63 ± 0.05 Bb	2.95 ± 0.20 Ab	24.99 ± 0.74 Aa	82.36 ± 5.12 Aa	376.88 ± 37.21 Aab	0.10 ± 0.03 Ab	6.62 ± 1.01 ABa	50.19 ± 5.17 Ba
	40–60	4.69 ± 0.02 Bb	2.79 ± 0.15 ABa	20.65 ± 1.39 Ba	80.81 ± 5.24 ABa	285.07 ± 26.47 Bab	0.07 ± 0.04 Ab	5.01 ± 0.62 Aa	36.29 ± 3.02 Cb
Hollow	0–20	4.71 ± 0.02 Ac	2.92 ± 0.21 Ba	20.05 ± 1.37 Ba	79.72 ± 5.53 Ba	258.05 ± 33.19 Ca	0.11 ± 0.02 Ab	9.81 ± 1.32 Aa	58.26 ± 5.91 Aa
	20–40	4.57 ± 0.07 Bb	3.41 ± 0.23 Aa	25.01 ± 1.53 Aa	93.09 ± 6.34 Aa	423.36 ± 39.75 Aa	0.09 ± 0.03 Bb	7.25 ± 1.07 Ba	57.10 ± 5.72 Aa
	40–60	4.63 ± 0.05 ABb	2.96 ± 0.20 ABa	22.33 ± 1.41 ABa	86.35 ± 5.74 ABa	336.13 ± 34.32 Ba	0.06 ± 0.01 Bb	5.32 ± 0.87 Ba	41.35 ± 4.28 Ba

Note: Data are given as mean ± standard deviation. Different capital letters indicate significant differences among three soil depths in the same microtopographical position (*p* < 0.05), and different lowercase letters indicate significant differences among three microtopographical positions at the same soil depth (*p* < 0.05).

## Data Availability

Data are contained within the article.

## Supplementary Materials

The following supporting information can be downloaded at: https://www.mdpi.com/article/10.3390/microorganisms12050867/s1, Figure S1: Rarefaction curves for all samples; Table S1: Summaries of sequencing rRNA library.
