# Supplementary material for: Effects of Microtopography on Soil Microbial Community Structure and Abundance in Permafrost Peatlands
Source: Microorganisms. 2024 Apr 26;12(5):867. doi: 10.3390/microorganisms12050867 (PMC11124213; doi:10.3390/microorganisms12050867)
Supplement: Supplementary file 1 [file microorganisms-12-00867-s001.zip › microorganisms-2977974-supplementary.pdf]

Supplementary Material

Table S1. Summaries of sequencing rRNA library.

	Samples	Raw reads	Clean reads	Effective reads	Mean length(bp)
Bacteria	27	1039821	1027584	615579	416
Fungi	27	1346093	1220376	797135	261

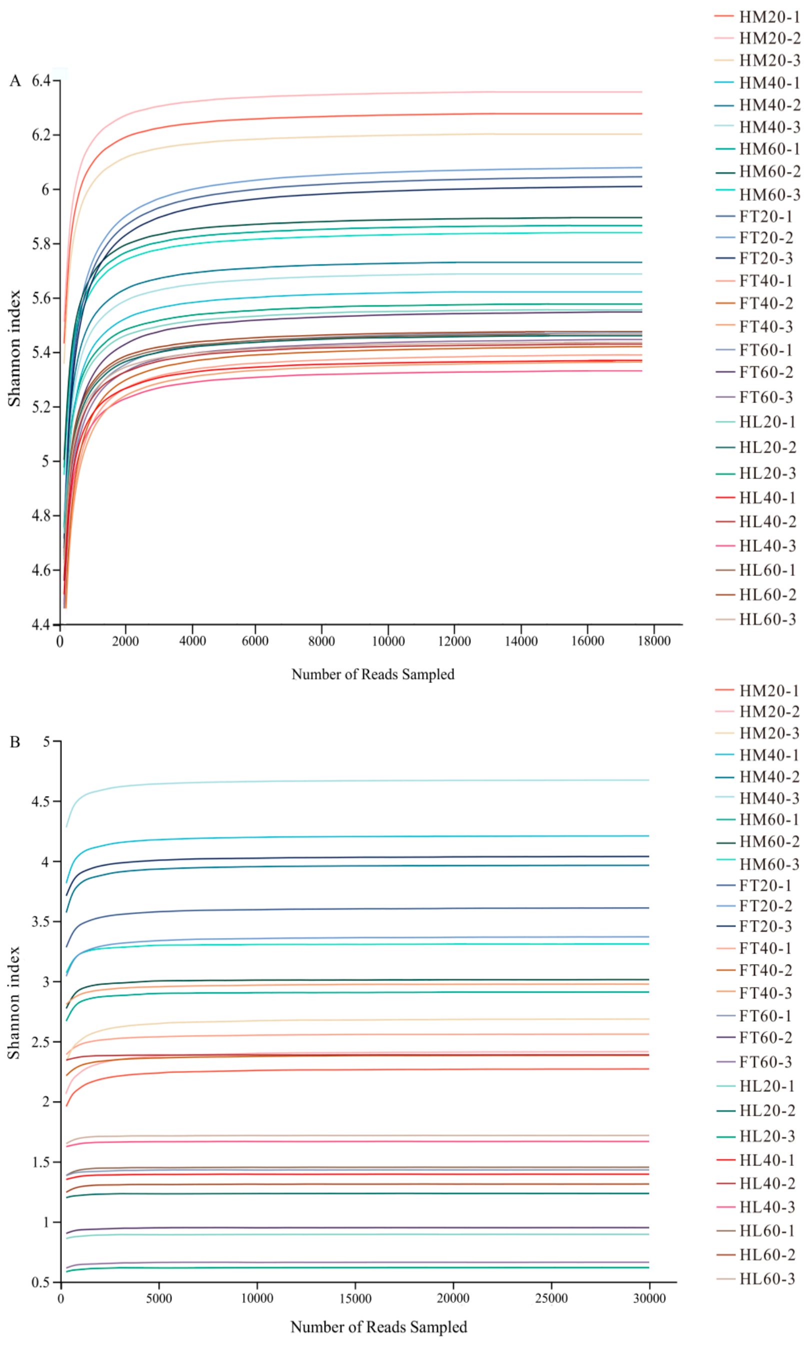

Figure S1. Rarefaction curves for all samples: (A) curves for bacterial sequences; (B) curves for fungal sequences.
